# Time-resolved searchlight analysis of imagined visual motion using 7 T ultra-high field fMRI: Data on interindividual differences

**DOI:** 10.1016/j.dib.2016.02.071

**Published:** 2016-03-05

**Authors:** Thomas C. Emmerling, Jan Zimmermann, Bettina Sorger, Martin Frost, Rainer Goebel

**Affiliations:** a Department of Cognitive Neuroscience, Maastricht University, 6229 EV Maastricht, The Netherlands; b Maastricht Brain Imaging Center, 6229 ER Maastricht, The Netherlands; c Netherlands Institute for Neuroscience (NIN), 1105 BA Amsterdam, The Netherlands

## Abstract

Interindividual differences play a crucial role in research on mental imagery. The inherently private nature of imagery does not allow for the same experimental control that is possible in perception research. Even when there are precise instructions subjects will differ in their particular imagery strategy and, hence, show different brain activations. Here, we show results of a time-resolved searchlight analysis for 12 individual subjects who perform a visual motion imagery task. The data show the spatial and temporal extent of brain areas and time windows that allow for a successful decoding of the direction of imagined motion out of four options. Accuracy maps for six different time windows are shown for every individual subject and are made freely available on NeuroVault. These data accompany the findings in the publication “Decoding the direction of imagined visual motion using 7 T ultra-high field fMRI” (Emmerling et al., 2016) [1].

**Specifications Table**

**Table:** 

Subject area	*Psychology*
More specific subject area	*Cognitive Neuroscience*
Type of data	*Maps*
How data was acquired	7 *T functional magnetic resonance imaging*
Data format	*Analyzed*
Experimental factors	*Significance maps from searchlight multi-voxel pattern analysis* (*MVPA*) *that were transformed into MNI-space*
Experimental features	*Decoding of four different directions of imagined visual motion throughout the cortical ribbon in* 12 *subjects*
Data source location	*Maastricht, Netherlands*
Data accessibility	*Data is within this article*

**Value of the data**

• Time-resolved significance maps show spatial and temporal differences between 12 subjects.
• Additional behavioral data from the original publication provide information about individual strategies and performance in widely used imagery questionnaires.
• Maps are available on NeuroVault.org making comparisons with other studies easier.

## 1 Data

We show data of a time-resolved searchlight analysis for 12 individual subjects performing a visual motion imagery task. In each searchlight sphere a multi-voxel pattern analysis (MVPA) is performed to decode four different directions of imagined motion. This is done for six different but overlapping time windows with respect to the onset of an imagery trial. The resulting volumetric maps show p-values for FDR-corrected Chi-square tests of the confusion matrices of each searchlight sphere. Maps were uploaded to NeuroVault.org (http://neurovault.org/collections/961).

## 2 Experimental design, materials and methods

### 2.1 Subjects

15 healthy fMRI-experienced subjects (six females; age: 27.4±6.3 years) volunteered in the original study [1]. Data from 12 subjects were analyzed based on exclusion criteria described in Emmerling et al. [1].

### 2.2 Procedure

Subjects attended three training sessions and one scanning session. The training was designed to practice the experimental task and more specifically a stable visual motion imagery. Details on the training procedure [p. 62; 1], the individual used imagery strategies [Table 1; 1], and behavioral results from the training sessions [Fig. 5; 1] are described in Emmerling et al. [1].

Subjects were pseudo-randomly assigned to one of two groups and had to imagine dots that moved in one of four directions (group 1: left, right, up, and down; group 2: four diagonal motion directions).

### 2.3 Additional behavioral data

All subjects filled in the Vividness of Visual Imagery Questionnaire [VVIQ; 2] and the Object-Spatial Imagery and Verbal Questionnaire [OSIVQ; 3]. For procedural details, analyses, and results please see the original publication [Figs. 3 and 4; 1].

For information on the experimental task, MRI acquisition, and imaging data preprocessing please refer to the corresponding sections in the original publication [p. 62–65; 1].

### 2.4 Searchlight analysis

Each experimental run was z-scored to eliminate signal offsets and variance differences between runs. The data were split into training and testing datasets. We employed a leave-one-run-out splitting procedure to be able to cross-validate the classification performance. To assess the spatial distribution of brain areas involved in the mental imagery task we performed a searchlight analysis [4]. A sphere with a radius of 4 voxels was moved through the cortical ribbon (so that the spheres central voxel always lay within −1 mm to +3 mm from the grey/white matter segmentation border) and defined a feature set of 257 voxels (4 voxel radius). For each voxel within each sphere and each trial we extracted the average of 6 s (3 TRs) as features. Averages were computed in six different but overlapping time windows starting at −2, 0, 2, 4, 6, and 8 s (*i.e.* −1, 0, 1, 2, 3, and 4 TRs) with respect to the trial onset. The extracted features were then used for an one-vs-one 4-class classification (predicted classes were chosen based on the maximum number of votes in all binary classifications) using a linear support vector machine [SVM; LIBSVM implementation in PyMVPA; 5]. The resulting classification accuracies were tested for significance by means of FDR-corrected Chi-square tests of the confusion matrix.

### 2.5 MNI space transformation

Individual anatomies were transformed to MNI-ICBM 152 space [6] using BrainVoyager 20.0 and the ICBM 452 template [7]. The individual significance maps for the six different time windows were resampled to a resolution of 1×1×1 mm^3^ voxel size and transformed into MNI space using the individual transformation matrices obtained in the previous step. The resulting maps were named according to the subject (“S01” through “S12”) and the time window (“_shift_−1” through “shift_4”) and uploaded to NeuroVault.org (http://neurovault.org/collections/961/).
